# Factors Associated with Providers’ Perceptions of Mental Health Care in Santa Luzia’s Family Health Strategy, Brazil

**DOI:** 10.3390/ijerph13010033

**Published:** 2015-12-23

**Authors:** Angela R. Ghesquiere, Rogerio M. Pinto, Rahbel Rahman, Anya Y. Spector

**Affiliations:** 1 Brookdale Center for Healthy Aging, Hunter College of the City University of New York, 2180 Third Ave, New York, NY 10035, USA; 2 School of Social Work, University of Michigan, Room 3792 SSWB, 1080 S. University Ave., Ann Arbor, MI 48109, USA; ropinto@umich.edu; 3 School of Social Work, Columbia University, 1255 Amsterdam Avenue, New York, NY 10027, USA; rr2640@columbia.edu; 4 New York City Department of Health and Mental Hygiene, 42-09 28th St, Long Island City, NY 11101, USA; aspector@health.nyc.gov

**Keywords:** transdisciplinary collaboration, mental health care, community health workers

## Abstract

Brazil has a unique mental health care system, characterized by universal coverage delivered by interdisciplinary teams both in the community and in specialized *centros de atenção psicossocial* (CAPS—psychosocial care centers). Provision of patient-centered mental health care is an important principle of Brazilian mental health care, but this topic has not been well-studied. We analyzed data from a cross-sectional survey of 151 community health workers (CHWs), nurses, and physicians in Santa Luzia, Minas Gerais State, Brazil. Chi-squares, *t*-tests and multivariate regression analyses examined differences in socio-demographics, caseload, engagement in evidence-based practices (EBPs), and transdisciplinary collaboration between providers who reported providing high levels of patient-centered mental health care and those who did not. In multivariate regression models, components of transdisciplinary collaboration were significantly associated with providers’ perceptions of patient-centered mental health care (*p* < 0.05). CHWs were also significantly more likely to report providing patient-centered care than physicians and nurses. EBP engagement and sociodemographics were not associated with perceptions. Results suggest that training efforts to improve patient-centered mental health care in Brazil could build upon CHWs’ skills and focus on transdisciplinary collaboration. Findings may inform practice in other countries with similar health care systems.

## 1. Introduction

Data from the World Health Organization (WHO) indicate that mental health disorders account for 12% of the global burden of disease [1]. Mental health disorders appear to be particularly prevalent in Brazil, a highly populous, diverse nation comprised of 26 states, one federal district and 5560 cities. A recent representative cross-sectional household sample of 5037 adults in one of Brazil’s largest cities, São Paulo, found that 29.6% of respondents reported having a mental health disorders in the last 12 months [2]. Anxiety disorders were the most common condition (19.9%), followed by mood (11.0%), impulse-control (4.3%), and substance use (3.6%) disorders [2]. These prevalence rates are slightly higher than in the U.S. (where 26.2% report mental health disorders in the last year) and about two times the estimates seen in other upper-middle income countries [3]. However, in Brazil, of those with severe mental health symptoms in the last 12 months, only 32.8% received any mental health services for their symptoms [2].

The Brazilian government has made efforts to improve mental health care in the last two decades, and now allocates more of its health budget on mental health than most countries (almost 3% of the national health budget) compared to about 1% in other developing countries [4]). Mental health care in Brazil is free and universal, as it is a part of the *Sistema Único de Saúde* (Unified Health System). A key component of the Unified Health System is integration of primary care and public health via the *Estratégia Saúde da Família* (ESF; Family Health Strategy). Established in 1994, the ESF led to the creation of transdisciplinary teams, where a range of providers (primarily nurses, physicians, and community health workers (CHWs) serve communities [4,5]. CHWs play an especially key role, acting the front-line providers in health education and disease prevention, including vaccinations and care of infants, assistance with medication administration, blood pressure screenings, and helping patients obtain and prepare healthy foods [5,6]. Most CHWs are low-income women, recruited because they have local knowledge their communities’ needs and norms geographies, cultural norms, and health resources and needs [5,7,8].

The ESF utilizes these health teams in an attempt to connect poor and socially vulnerable communities to primary health care and is centered on a family and community approach. Community members are referred to universal mental health care via primary care services; health workers help identify mental health needs and then make linkages to mental health services [4,9]. Mental health care in Brazil is centered around community mental health centers, called *Centros de Atenção Psicosocial* (CAPS) [4,10]. CAPS provide both intensive outpatient care and brief partial hospital care to community residents with severe mental health disorders [11].

The entire Brazilian system is intended to address patient needs in a “patient-centered” manner, so as to make care more effective and efficient [10]. “Patient-centered care” is that in which providers listen to patient needs and share decision-making, rather than acting solely on what the provider thinks is best for the patient [12,13,14]. The concept has received a great deal of research and clinical attention in recent years [12,13]. Components of patient-centered care include care which explores the patients’ primary concerns, answers patient questions about care, seeks an integrated view of the patient which takes their world view into account, finds common ground between the patient and provider on what the problem is, agrees upon the solution to the problem, and encourages a continuing relationship between patient and provider [12,15]. Research from nations as diverse as Italy, the U.K., the U.S., Canada, South Africa, and Norway indicate that patients desire such care [12,15]. Moreover, patient-centered care has been associated with a number of positive outcomes, including high patient satisfaction, treatment adherence, and improved health [16]. Though most literature in this area has focused on health care, researchers have recently recommended attention to patient-centered care in mental health care [17,18], and one recent paper explored what patient centered mental health care might entail for Brazilian immigrants in the U.S. [19]

Despite the novel structure of the Brazilian system and its’ cutting-edge focus on patient-centered care, only a few studies have examined Brazil’s mental health apparatus. Previous studies have described mental health policy, characterized the current mental health system [4,9,11], and described the experience of mental health patients, family members, and service providers within Brazil’s mental health system [20]. We were unable to identify any studies that examine providers’ sense of whether they were providing patient-centered mental health care, however.

Our study, thus, addresses this gap in the literature by examining factors (specifically, socio-demographics, employment-related factors, trandisciplinary collaboration and engagement in evidence-based practice) that might be associated with providers’ perceptions of provision of patient-centered mental health care.

## 2. Experimental Section

### 2.1. Methods

U.S.- and Brazil-based researchers and ESF staff developed a partnership in order to conduct this study [21,22]. The team developed a multidimensional survey grounded in in-depth interviews with ESF staff. Two Master’s level Brazilian interviewers administered cross-sectional surveys using password-protected mobile computers. Data were downloaded into and managed in a password-protected database (Illume 4.6; DatStat; Seattle, WA, USA [23]); Interviews were all conducted in Portuguese, and lasted 45 to 75 min. We gave participants information about their rights, risks and benefits, and confidentiality. Participation was voluntary; providers were offered refreshments during surveys. The sample included 151 ESF workers (physicians, nurses and community health workers) in Santa Luzia, Minas Gerais State (for complete description of procedures see [22]). Santa Luzia was selected for the mental health survey in part because they were one of the first municipalities in Brazil to devote substantial resources to mental health care and because the U.S.-based Principal Investigator had a working relationship with ESF administrators.

### 2.2. Measures

The complete survey included questions on providers’ sociodemographics, and opinions on transdisciplinary collaboration and evidence-based practice (EBP) engagement. Surveys were pilot tested with, and modified based on feedback from, with CHWs, nurses, and physicians, as each group had different reading comprehension levels and preferences for survey length. Study staff translated all survey questions into English and then back-translated them to Portuguese, using standard protocol [24].

### 2.3. Dependent Variable

Providers’ perceptions of patient-centered mental health care provision was measured by a composite of three items, with all items created for this study: How often the provider listened to what the patient things about their mental health disorder, how often the provider listened to what the patient thinks about their treatment, and how often the provider listened to what the patient thinks is related to their mental health or disorder. Providers were asked to rate how often they demonstrated each listening behavior (Always, In Most Cases, Less than Half the Time, Rarely, and Never), out of a possible range of 3–15. The three items had good internal consistency (Cronbach α = 0.70). The composite was then dichotomized at its median value (11, out of an actual range of 3–12), to allow for comparison of those who showed a trend towards providing patient-centered mental health care and those who did not.

### 2.4. Independent Variables

Socio-demographics—Participant age was measured in years. Race was self-identified as White, Black, or Pardo (a broad classification encompassing mixed races, mulattos, and indigenous people). Gender was categorized as male or female.

Employment-related factors—Providers were physicians, nurses, or CHWs. We did not separately include an education variable because of its overlap with staff categories; all physicians had advanced degrees, all nurses had high school educations, while most CHWs only completed junior high school. Length of time in position was measured by a single variable: “How long have you worked for the ESF?” (in months). Caseload was also measured by a single variable: “How many families do you serve?” One outlying response (10,000 families served) was removed from analyses, as it inflated standard deviations considerably.

Transdisciplinary collaboration—Transdisciplinary collaboration is characterized by providers working together to incorporate various types of knowledge, and identifying solutions by transcending disciplinary perspectives [25]. In the Unified Health System, multi-professional teams work under the principle of “comprehensive care” [26,27]. The CHWs on ESF teams provide home visits to residents in impoverished neighborhoods, often the same communities in which they reside, with high prevalence of chronic diseases (e.g., diabetes, hypertension) and help to connect individuals to a variety of services to meet their needs [28], including health counseling and education, identifying individuals in need of ongoing care and/or monitoring for chronic health conditions [29], and social support services, such as access to food vouchers and child welfare services [30,31,32]. Consultations with specialists, such as pharmacists, psychiatrists, and dentists, are also built into the system, though the availability of specialists varies widely across municipalities [31].

Consistent with previously published analyses [22], we measured transdisciplinary collaboration with several variables. Importance of team participation was a two-item composite (Cronbach α = 0.73) “Weekly team meetings are important for discussing patients’ problems” and “Weekly team meetings are important for training and treatment planning.” Participants gauged their agreement with survey statements using a 5-point Likert scale. We also measured transdisciplinary mental health care with two individual items: “Your opinions about mental health patients under your care are considered by the team” and “Your team tries to engage other sectors, services, and resources in planning treatment.” (Always, In most cases, Less than half the time, Rarely, and Never).

Integration of Evidence Based Practice (EBP)—All ESF providers are encouraged through training provided by government agencies to utilize evidence-based practice (EBP). EBP involves consciously using the current the best available research evidence when making decisions about patient case, and integrating this evidence with clinical expertise and consideration of patient values and preferences [33]. EBP can also be thought of as the process by which providers use assessment and treatment that is based on research findings [14]. In Brazil, EBP is encouraged by training local providers in methods to integrate information from multiple sources, including clinical experience, existing research evidence, primary care provider reports, family member reports, and client reports [34]. We do not focus on individual EBPs, but examine providers’ general use of research findings in care provision. Consistent with previously published analyses [22], we measured integration of evidence-based practice using a composite of five items: “I use research findings to help patients”, “I can find the information I need to help patients”, “I know how to use new information to change the way I treat patients”, “I am able to understand and use protocols to help patients”, “I combine information for different sources to address patients’ needs.” Items were all scored on a 5-point Likert Scale (Strongly Agree, Agree, Neither, Disagree, and Strongly Disagree). Internal consistently of these items was acceptable (Cronbach α = 0.57).

### 2.5. Data Analysis

After conducting descriptive statistics, we use Chi-squares and *t*-tests in order to examine differences in sociodemographic factors, employment factors, transdisciplinary collaboration, and use of EBP between providers who reported providing high levels of patient-centered mental health care and those who did not.

We next conducted multivariate logistic regression models to test if factors that were significant in preliminary analyses remained associated with high patient-centered care provision.

IBM SPSS Statistics Version 19 was used to carry out all analyses, and regression models were also calculated in SAS (version 9.1; SAS Institute Inc., Cary, NC, USA) to confirm the accuracy of results. Given the dearth of existing literature in this particular area and the relatively small sample size, we considered *p*-values ≤ 0.10 as statistically significant.

## 3. Results

Of the 151 Santa Luzia participants, the majority (*n* = 131; 86.8%) were female and most were CHWs (*n* = 92; 69.9%). Thirty-nine (25.8%) were nurses while 20 (13.2%) were physicians. Most (*n* = 91, 60.3%) identified their race as Pardo, while 38 (25.2%) identified as White and 22 (14.6%) as Black. Average participant age was 33.60 (Standard Deviation (SD) = 9.99, range = 20–70). These sociodemographic factors were similar to those in the entire (two-site) study sample, as presented previously [22].

### 3.1. Bivariate Results

Using a median split of the composite variable scores, 100 participants were categorized as perceiving that they were providing patient-centered mental health care and 51 as not providing patient-centered mental health care. As shown in Table 1, no sociodemographic factors were associated with perception of patient-centered mental health care provision. Two employment factors were, however: those who thought they were providing patient-centered mental health care were more likely to be CHWs (69%) than nurses (20%) or physicians (11%; χ^2^(1) = 8.19, *p* < 0.05), and had lower case-loads than those who did not (697 *vs.* 1091, χ^2^(1) = 8.19, *p* < 0.05). It should also be noted that caseload was highly associated with role, with CHWs having significantly lower caseloads (M = 256.63, SD = 152.11) than nurses (M = 1803.16, SD = 1369.24) or doctors (M = 1603.75, SD = 739.77), F = 69.46, *p* < 0.001. This reflects the structure of the ESF, in which CHWs are the primary contacts for families and physicians and nurses consult on a large number of cases.

**Table 1:** Predictors associated with providers’ perceptions of patient-centered mental health care provision (*n* = 151).

Variables	High Patient-Centered Care (*n* = 100)	Low Patient-Centered Care (*n* = 51)	Test Value, *p*-Value
Socio-demographics:	*n* (*%*)	*n* (*%*)	
Female (*n* = 131)	87 (87.0)	44 (86.3)	0.15, *p* = 0.901
Race			0.58, *p* = 0.749
White (*n* = 38)	24 (24.0)	14 (27.5)	
Black (*n* = 22)	16 (16.0)	6 (11.8)	
Pardo (*n* = 91)	60 (60.0)	31 (60.8)	
	*M* (*SD*)	*M* (*SD*)	
Age (years)	33.26 (10.17)	34.25 (9.70)	0.58, *p* = 0.565
	*n* (*%*)	*n* (*%*)	
Employment factors:			
Provider			8.19, *p* = 0.017
CHW (*n* = 92)	69 (69.0)	23 (45.1)	
Nurse (*n* = 39)	20 (20.0)	19 (37.3)	
Doctor (*n* = 20)	11 (11.0)	9 (17.6)	
	*M* (*SD*)	*M* (*SD*)	
Number of families served at position	696.68 (847.30)	1091.63 (1302.74)	2.23, *p* = 0.027
Length at position (months)	49.10 (37.07)	29.45 (39.38)	0.06, *p* = 0.956
Transdisciplinary Collaboration			
Importance of team meetings (composite)	7.12 (1.01)	7.33 (0.97)	1.24, *p* = 0.215
Tries to engage other sectors in treatment planning	2.97 (1.10)	2.51 (1.16)	−2.31, *p* = 0.022
Opinions about mental health are considered by the team	3.58 (0.71)	3.22 (0.64)	−3.07, *p* = 0.003
Use of Evidence Based Practices (composite)	12.73 (2.64)	12.35 (2.86)	0.81, *p* = 0.421

In unadjusted analyses examining components of transdisciplinary collaboration, importance of team meetings was not associated with provision of patient-centered mental health care; however, trying to engage other sectors in treatment planning (M = 2.97 *vs.* 2.51; *t* = −2.31, *p* < 0.05) and having ones opinions frequently considered by the team (M = 3.58 *vs.* 3.22; *t =* −3.07, *p* < 0.01) were. The composite item of EBP use was not associated with perception of provision of patient-centered mental health care.

### 3.2. Regression Results

In multivariate regression models which included all significant independent variables, trying to engage other sectors in treatment planning and helping clients meet their own needs and engaging other sectors in treatment planning remained significant (*p* < 0.05) (Table 2). CHWs were also significantly more likely to believe that they were providing patient-centered care. Caseload was no longer significantly associated with perception of provision of care, however, likely because of collinearity between provider role and caseload. Other variables were not significant in exploratory analyses and were dropped from the final model.

**Table 2:** Logistic regression reporting factors associated with providers’ perceptions of patient-centered mental health care provision (*n* = 151).

Effect	Beta	SE	Chi-Square	*p*-Value
**Employment factors:**				
CHW	1.376	0.510	3.957	0.007
Number of families served at position	0.000	0.000	0.674	0.412
**Transdisciplinary Collaboration**				
Importance of team meetings (composite)	−0.232	0.223	1.073	0.300
Tries to engage other sectors in treatment planning	0.381	0.171	4.936	0.026
Opinions about mental health are considered by the team	0.981	0.314	9.749	0.002
**Use of Evidence Based Practices (composite)**	0.099	0.072	1.894	0.169

OR = Odds Ratio; CI = Confidence Interval.

## 4. Discussion

This study is unique in its focus on provision of patient-centered mental health care in Brazil’s Unified Health System. This appears to be the first study to examine factors associated with providers’ perception of provision of patient centered mental health care in Brazil. Provider role and components of transdisciplinary collaboration seem especially highly associated with perception of provision of patient-centered mental health care. This study is a first step in better understanding factors associated with quality mental health care in Brazil, and may have implications for similar practices elsewhere, as it provides insight into factors that influence provision of patient-centered care. Results are also consistent with previous studies of patient-centered medical care in the U.S., which found that provider discipline was associated with attitudes to patient-centered care provision [35]. Though the health care systems are quite different between the U.S. and Brazil, the notion that different providers types may have different perceptions of the care they are providing is likely consistent.

The findings point to several modifiable factors that might improve the use of patient-centered mental health care in Brazil. Our finding that CHWs are most likely to believe they are providing patient-centered care suggest that there may be CHW skills, which make them better able to communicate well with their clients. CHWs are usually drawn from local communities, and are already familiar with local geography and norms [5,7,8]. Pinto and colleagues have proposed that CHWs in Brazil use a combination of indigenous strategies (empathic communication and perseverance) combined with specific technical skills in health promotion and disease prevention to work effectively with patients [5].

The lower case load of CHWs also likely provides them with adequate time and energy to have these discussions. Previous research indicates that CHWs’ skills are often shared with the other providers in the team and can enhance ESF communication with low-income community members [36,37].

Collaboration among ESF team members also seems highly associated with provision of patient-centered care. Transdisciplinary ESF care in Brazil has been found to be effective in reducing HIV transmission rates, increasing immunization rates, and reducing infant and child mortality [27,31,38,39]. Though the exact mechanism for these outcomes has not been identified, it is possible that improved communication, improvement in attitudes towards other professions, fostered professional interactions, and application of knowledge from a range of disciplines leads to improvements in quality of patient care [22,25].

There may be several ways to enhance transdisciplinary collaboration, which could be adapted and implemented in Brazil. These include applying common theories and mechanisms of behavior change, (such as the Theory of Reasoned Action and Planned Behavior Theories [40,41]) in designing change, and utilizing knowledge from different practitioners in the development of training curricula and materials [42]. Other recommendations which might potentially improve collaboration include strong leadership support for these efforts, supervision and peer support to staff involved in making linkage, and provision of adequate office accommodation conducive to collaboration [43]. Recently, researchers conducted to a training meant to enhance collaboration between primary care and mental health providers in Brazil, in which different disciplines (e.g., primary care doctors, social workers, nurses, psychologists, and psychiatrists) attended training together, learned communication skills, and used active learning techniques like role plays. The study found significant increases in the provision of patient-centered care post-training [44]. These efforts could be more widely disseminated.

Previous studies have also found differences in patient-centered behavior by provider gender, with women more likely to provide patient-centered care than men [45,46]. We did not identify this trend, but our sample was predominantly female, reducing power to detect any gender differences. Additionally, our finding that use of evidence-based practices was not associated with patient-centered care implies that other traits and skills are more important in influencing this behavior.

### 4.1. Limitations

The current study has several limitations. First, data were only collected on provider perceptions, not on client or family perceptions. It is possible that provider perceptions could be biased, and not reflective of the care provided. Second, we only collected self-report data, increasing the chances of bias and measurement errors. An additional measurement limitation is that our dependent variable was created for this study, and the cut-off created based on the median of the current sample. The validity of the dependent variable is not well-established, and findings may not generalize to other samples.

Third, data were only collected cross-sectionally, making it impossible to determine the directionality of influences among the variables we examined. For example, it is possible that providing patient-centered care leads to enhanced transdisciplinary collaboration, and it is also possible that positive transdisciplinary collaboration leads to more patient-centered care provision. A longitudinal design would have allowed for a more comprehensive understanding the associations between variables of interest and perceptions of patient-centered mental health care provision.

Using this dataset, we are unable to examine whether provider perceptions of patient-centered mental health care provision is associated with any differences in patient outcomes (e.g., satisfaction, symptom reduction, re-hospitalization). In the U.S., patient-centered care has been associated with better patient health outcomes [16], and it remains to be seen whether these associations also occur in Brazil. Finally, the data were collected in only one city in Brazil. There are vast disparities in mental health resource distribution across Brazil geographically [4,11]. Generalizability of findings is therefore limited.

### 4.2. Areas for Future Research

Future research could gather data longitudinally and examine how actual services provided are associated with various patient outcomes, such as reductions in mental health symptoms or satisfaction with care. Future studies could also collect data on client or family perceptions of mental health care. Client-level data would more accurately capture whether care provided is actually patient-centered. Moreover, previous research has identified some suggestions for improvement in mental health care provision among community residents and family members in Brazil [20]; the specific impact of patient-centered mental health care on community residents and their family members could also be studied. The association between number of visits and patient-centered mental health care could also be examined, as well as how both patients and provider perceive the benefits and drawbacks (e.g., time burden) of multiple visits. Replication of findings outside of the single city studied here is also important. Future studies could also collect more detailed data on mental health care provision and transdisciplinary collaboration between health and mental health.

It should also be noted the CHWs likely have very different roles in providing mental health care that do nurses, psychologists, or psychiatrists. CHWs may be most likely to assist with medication adherence and connect patients to services, while nurses, psychologists and psychiatrists would provide more intensive psychopharmalogical or therapeutic interventions [44]. The ways that providers’ distinct roles could affect care provision should also be studied. Future efforts will be needed to identify how training can improve all workers’ skills in providing patient-centered mental health care. This might include techniques like peer mentorship, training in assessment and communication, and increase in the number of team meetings.

## 5. Conclusions

The current study fills an important gap in the research literature by providing needed data on mental health care provision in Brazil and identifying factors that might influence the provision of patient-centered mental health care. Future research could collect more data on outcomes of providing patient-centered mental health care, and seek more input from community residents and their family members.
